# Modelling and Analysis of Hydrodynamics and Water Quality for Rivers in the Northern Cold Region of China

**DOI:** 10.3390/ijerph13040408

**Published:** 2016-04-08

**Authors:** Gula Tang, Yunqiang Zhu, Guozheng Wu, Jing Li, Zhao-Liang Li, Jiulin Sun

**Affiliations:** 1 State Key Laboratory of Resources and Environmental Information Systems, Institute of Geographical Sciences and Natural Resources Research, Chinese Academy of Sciences, Beijing 100101, China; sunjl@igsnrr.ac.cn; 2 The Engineering Science, Computer Science and Imaging Laboratory, The National Center for Scientific Research, University of Strasbourg, Illkirch 67412, France; lizl@unistra.fr; 3 University of Chinese Academy of Sciences, Beijing 100049, China; 4 Jiangsu Center for Collaborative Innovation in Geographical Information Resource Development and Application, Nanjing 210023, China; 5 Southern Trunk Channel Management Office of South-to-North Water Diversion Project, Beijing 100195, China; wgzh08@163.com; 6 Heilongjiang Provincial Research Institute of Environmental Science, Harbin 150056, China; lijing020801@126.com; 7 Key Laboratory of Agri-informatics, Ministry of Agriculture, Institute of Agricultural Resources and Regional Planning, Chinese Academy of Agricultural Sciences, Beijing 100081, China

**Keywords:** cold region, hydrodynamic, water quality, model, numerical simulation

## Abstract

In this study, the Mudan River, which is the most typical river in the northern cold region of China was selected as the research object; Environmental Fluid Dynamics Code (EFDC) was adopted to construct a new two-dimensional water quality model for the urban sections of the Mudan River, and concentrations of COD_Cr_ and NH_3_N during ice-covered and open-water periods were simulated and analyzed. Results indicated that roughness coefficient and comprehensive pollutant decay rate were significantly different in those periods. To be specific, the roughness coefficient in the ice-covered period was larger than that of the open-water period, while the decay rate within the former period was smaller than that in the latter. In addition, according to the analysis of the simulated results, the main reasons for the decay rate reduction during the ice-covered period are temperature drop, upstream inflow decrease and ice layer cover; among them, ice sheet is the major contributor of roughness increase. These aspects were discussed in more detail in this work. The model could be generalized to hydrodynamic water quality process simulation researches on rivers in other cold regions as well.

## 1. Introduction

Ice-covered rivers are a common natural phenomenon. In China, an ice-coverage phenomenon may occur every winter in the regions north of northern latitude 30° and on the Qinghai-Tibet Plateau. At higher latitudes, the possibility of freezing becomes higher with longer periods of freezing weather; especially in the northeast, the ice-covered period of rivers can be as long as 5 months. Ice-covered rivers may cause many adverse effects such as loss of river shipping, ice floods resulting from ice jams or ice dams and frost heaving damage to hydraulic structures [1,2]. At present, although various studies on ice-covered rivers have been carried out by researchers, most of them focus on the aspects of streams, ice sheet evolution and hydropower [3,4]. In fact, as an impact of ice covers, great changes in water quality characteristics as well as mixing ability and transportation, and dispersion characteristics and so on occur in ice-covered rivers. Hou *et al.* [5], Chambers *et al.* [6] and Terzhevik *et al.* [7] studied dissolved oxygen transformation rules of frozen watercourses, while Gotovtsev *et al.* [8] adopted an analytical solution of the modified Streeter-Phelps equations system to derive formulas for calculating the biochemical oxygen demand and the rate of biochemical oxidation in a water body or a river channel covered by ice. However, Sun *et al.* [9] simulated the 2005 nitrobenzene water pollution incident in the Songhua River by establishing a hydrodynamic water quality model. Oveisy *et al.* [10] illustrated the importance of ice cover for simulation of winter dynamics of DO and Chl-a in Lake Erie between the USA and Canada by constructing a model which could be used as a tool for the investigation of climate change effects and water quality in ice-covered lakes. Through surveys and simulations, Sugihara *et al.* [11] studied the water quality characteristics of stagnant, eutrophic water bodies that freeze in winter and examined how climate change might influence the water quality characteristics of ice-covered, stagnant, eutrophic water bodies.

In China, because of insufficient observations, it has been found that there are no comparative studies on the hydrodynamics and water quality processes of an identical river during its ice-covered and open-water periods. What are differences in the hydrodynamic process and the pollutant migration and transformation process of a watercourse during its ice-covered and open-water periods? What are reasons leading to such differences? What are main influencing factors or indicators? Those problems are very worthy of being studied. Therefore, it is fairly significant to carry out a comparative simulation analysis for water quality of rivers during their ice-covered and open-water periods in the northern cold region of China. For this study, the research purpose is to simulate transportation and dispersion processes of pollutants in ice-covered and open-water periods and to perform corresponding comparative analysis. Moreover, by constructing a typical hydrodynamic water quality model for rivers in northern cold region of China, transformation patterns under different climatic conditions are revealed. As a result, decision support information can be provided with mathematical model of water environment in the Mudan River watershed and other similar rivers.

## 2. Selection of Research Area and Simulated Pollutants

**Figure 1:**
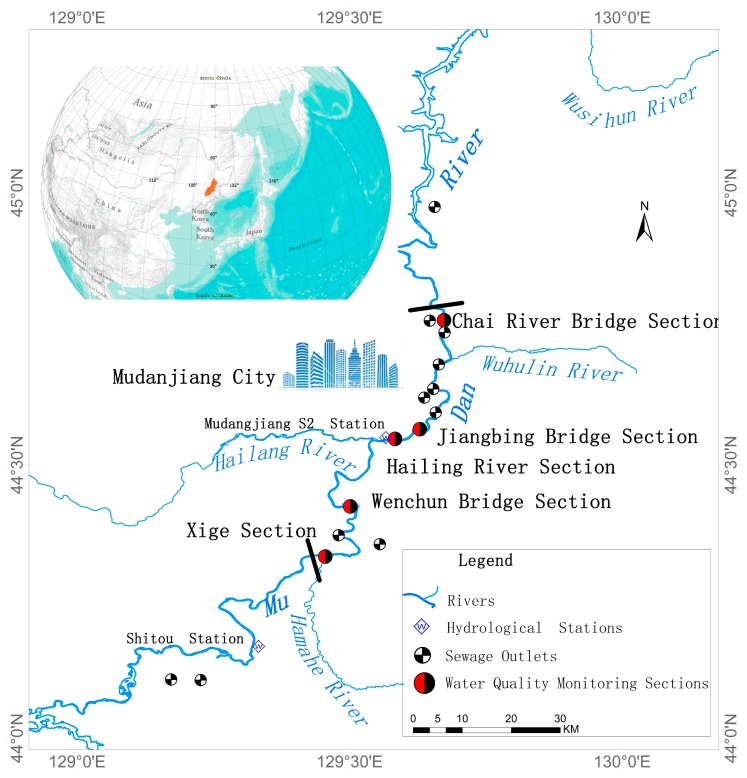
Schematic diagram for rivers, sewage outlets and monitoring sections in the research area.

The typical river selected in this work is the Mudan River in the northeastern region of China. Its basin perimeter ranges from northern latitude 42°54′ to 46°20′ and eastern latitude 127°34′ to 130°46′ (Figure 1). According to the meteorological monitoring data statistics from 1980 to 2010, the annual average temperature in the Mudan River watershed is 4.3 °C. Furthermore, its average temperature in January, which is the coldest time of the year, is −17.3 °C with an extreme minimum temperature of −38.3 °C. On contrary, the hottest time is July, when the average temperature reaches 22.3 °C and its extreme maximum temperature is 38.4 °C. With a total length of 726 km, the Mudan River has a total fall of 1007 m, an average gradient of 1.39‰ and a drainage area of 37,023 km^2^. At the mouth of Mudan River, the multi-year average discharge and runoff are 258.5 m^3^/s and 5.26 billion m^3^, respectively; in particular, its maximum runoff is up to 14.9 billion m^3^ [12]. As it is located in a cold region, Mudan River has a five-month long ice-covered period each year.

Multiple residential areas and industrial districts are distributed along the trunk stream of the Mudan River. Consequently, large amounts of industrial wastewater and sanitary sewage have been discharged into it, so its water quality is not desirable. Research results presented by Hao *et al.* [13] revealed that the water body in the urban section of the Mudan River trunk stream presents organic pollution features; chemical oxygen demand (COD_Cr_) and ammonia nitrogen (NH_3_N) are the primary factors exceeding the standard. Dissolved oxygen (DO) is also another important index of water quality in rivers [6,7], The concentration of DO in the Mudan River was in a range 6–11 mg/L within the standard according to the observed data, so just the COD_Cr_ and NH_3_N transportation and dispersion processes in the Mudan River were stimulated. In this study, the EFDC hydrodynamic water quality model is adopted to perform a new two-dimensional numerical simulation for transportation and dispersion processes of COD_Cr_ and NH_3_N in the Mudan River in the urban sections of its trunk stream.

## 3. Introduction to the Model

At present, several hydrodynamic water quality models have been widely applied, including Delft3D [14], WASP [15], MIKE11/21/3 [16,17,18], CE-QUAL-W2 [19] and Environmental Fluid Dynamic Codes (EFDC), *etc.* The EFDC model is a comprehensive dynamic tool for all feature-required studies [20].

As a multi-parameter finite difference model, it can simulate one-dimensional, two-dimensional, and three-dimensional hydrodynamics and water quality of water bodies in rivers, lakes, reservoirs, wetlands, river mouths, bays, and oceans. The model consists of six modules: hydrodynamics, water quality, toxic substances, substrate, stormy waves, and sediment. It was used to simulate processes such as surface water flow field, material transport (including water temperature, salinity, tracer agent, cohesive sediment, and non-cohesive sediment), and water body eutrophication [20].

EFDC model is regarded as a technically defensible 3-D hydro-environmental modeling tool for pollutant and pathogenic organism transport and fate from point and non-point sources, simulation of power plant cooling water discharges, simulation of oyster and crab larvae transport, and evaluation of dredging and dredge spoil disposal alternatives [21,22,23,24,25,26]. It is strongly recommended by U.S. EPA as a standard hydro-environmental simulation tool. Therefore, in view of the extensive application cases of EFDC, an attempt was made in this research to construct a water quality simulation model based on such a model and apply it to a hydrodynamic water quality simulation study of the ice-covered and open-water periods of the Mudan River trunk stream located in the northern cold region of China so as to provide a decision-making basis for watershed management.

The hydrodynamic equations in the EFDC model are based on a three-dimensional incompressible, graded-density turbulence boundary layer equation set, including the momentum equation, continuity equation, and material transport equation. The Boussinesq hypothesis [27] is often adopted to facilitate the processing of buoyancy lift terms caused by density contrast. The model uses either stretched or sigma vertical coordinates and Cartesian or curvilinear orthogonal horizontal coordinates. The two turbulence parameter transport equations based on the Mellore-Yamada level 2.5 turbulence closure schemes are used [28].

A second-order accuracy finite difference formulation is adopted in the solution of momentum equations and continuity equation. Staggered (or C) grid scatter is adopted horizontally [29]. Second-order accuracy finite difference in three time level scheme is adopted in time integration. The solution is divided into the internal mode and the external mode, *i.e.*, free surface gravity waves and shear stresses are solved in splitting methods [30]. Semi-implicit difference schemes are adopted in the solution in the external mode. Two-dimensional water level elevation is calculated simultaneously. In this mode, pre-processing is conducted in the conjugate gradient method before solution [31]. The solution method allows large-scale time step calculation. Time step is only constrained by the explicit central difference stability criterion or the high-order windward advection algorithm of the non-linear accelerating algorithm [32]. Implicit difference scheme with consideration of vertical diffusion is adopted in the solution to the internal model [33].

To minimize numerical diffusion, the multidimensional positive definite advection transport algorithm is adopted in the model [34,35]. The algorithm adopts first-order accuracy spatially and second-order accuracy temporally. More details of the governing equations and the numerical schemes can be found in Hamrick [20].

## 4. Model Construction

### 4.1. Simulation Range and Time Interval

Initial and terminal sections studied in this work are the Xige Section and the Chai River Bridge Section, respectively, covering a distance of 77.7 km. Hailang River, a main tributary of Mudan River, feeds into this river link, as shown in Figure 1. Considering that the river remains in a frozen state for five months every year, changes in sediment transport during its passage to ice-covered regime take place primarily due to changes in flow dynamics caused by the additional resistance at the upper boundary [36], and its hydrodynamic parameters, comprehensive decay rate of pollutants and roughness in ice-covered period are significantly different from each other due to impacts of ice sheets. Therefore, simulations performed by this model are separately carried out for the ice-covered and open-water periods. For the purpose of data integrity of this study, the simulation interval for the open-water period is from 1 January 2012 to 15 November 2014; that is 1050 days in total. With regard to the model itself, 1 January of 2012 is set as the first day, and the number of days mentioned below is counted by starting from this date as the start. Ice-covered and open-water periods within the simulation interval are listed in Table 1.

### 4.2. Grid Division and Boundary Condition Setting

#### 4.2.1. Grid Division

**Figure 2:**
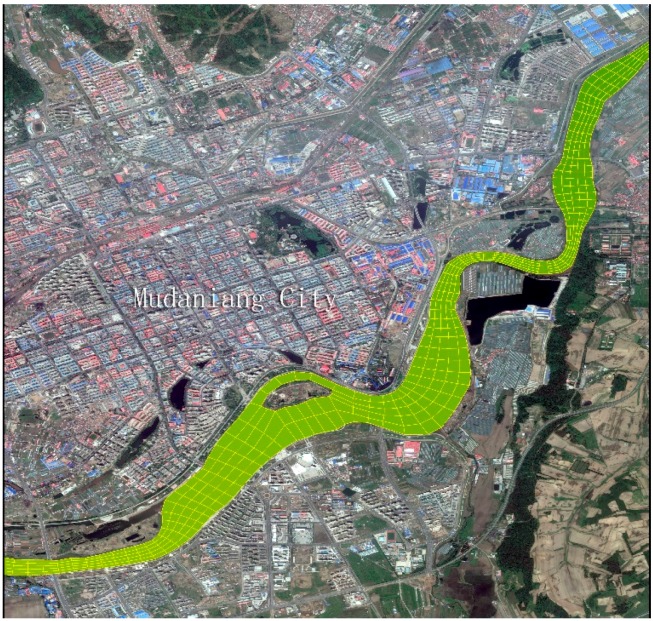
Grid division for urban section of the Mudan River trunk stream (part).

**Figure 3:**
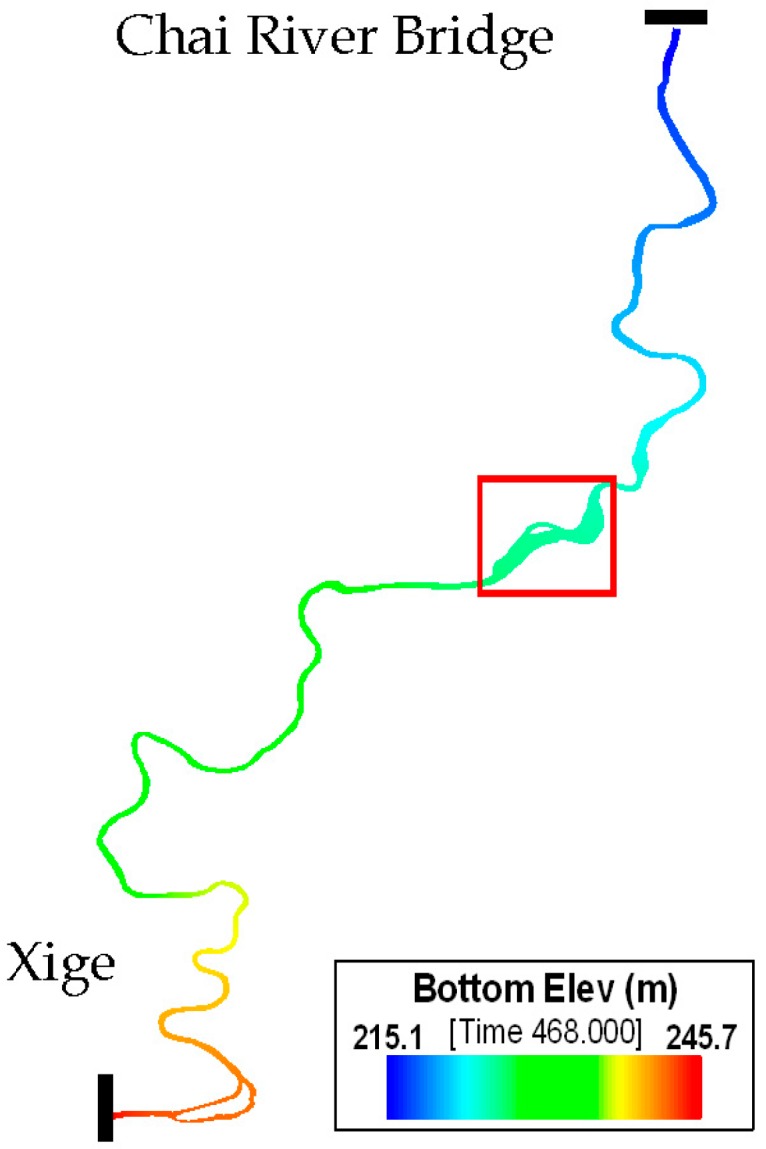
Bottom elevation diagram for urban section of the Mudan River trunk stream.

According to the actual terrain in the urban section of the Mudan River trunk stream, an orthogonal curvilinear grid is employed to divide its water body into 4207 cells with a scale ranging between 24 m × 43.6 m and 176.9 m × 241.3 m (Figure 2). In addition, the grid matrix is 864 lines × 5 columns. Bottom elevation of the simulated river section lowers down from 245.7 m on the Xige section to 215.1 m on the Chai River Bridge section (Figure 3). One vertical layer was used in the model simulation because of the shallow water in the Mudan River.

#### 4.2.2. Boundary Conditions

Boundary conditions of the simulated flow include upstream on-coming flow of the Xige section, downstream water discharge from Chai River Bridge section, flow from Hailang River fed into the trunk stream and quantity of wastewater effluent from sewage discharge outlets along the river. Within the simulated river section, as the only hydrology monitoring station named Mudan River Hydrological S2 Station (Figure 1) is established in the Hailang section and water quality monitoring stations are set on the Xige and Chai River Bridge sections, so the on-coming flows for the Xige and Hailang sections can be calculated on the basis of observed flow data in the Mudan River Hydrological S2 Station. According to the proportions taken by drainage areas controlled upstream of Xige Section and sub-stream of Hailang River, respectively, observed flow data from Mudan River Hydrological S2 Station are allocated to the Xige section and the mouth of Hailang River. Among them, the flow of the former represents 2/3 of that measured at Mudan River Hydrological S2 Station, while the flow of the latter feeding into the Mudan River accounts for 1/3 in that of such a station. Concerning the boundary conditions of Chai River Bridge, the open boundary condition which is also known as the water level condition is adopted. Corresponding water level data can be acquired by using the observed water level at Mudan River Hydrological S2 Station minus the difference between the bottom elevation of the section and such an elevation of the Chai River Bridge section. It should be noted that pollution discharge at the sewage outlets of the trunk stream is observed flow data.

Boundary conditions for concentrations incorporate pollutant concentrations in upland water from upstream of the Xige section, in inflows from Hailang River and in all sewage outlets along the river. Four water quality monitoring sections, namely Wenchun Bridge, Hailang, Jiangbin Bridge and Chai River Bridge are model verification sections. Water quality monitoring in sections is carried out in January, February, May, June, July, August, September and October of each year; among the results, January and February represent the water qualities during the ice-covered period, while the remaining months stand for those of the open-water period. Within the trunk stream simulation section, 11 main sewage outlets are covered and their positions are shown in Figure 1.

According to the measured data, there is no continuous flow monitoring data from these 11 sewage discharge outlets, and only the total pollutant discharge of the whole year is available. Consequently, a constant value is adopted for the flow of those outlets. As the concentration of pollutants is monitored quarterly at each outlet, observed values are employed for boundary conditions of concentrations. Every year, approximately 60 million m^3^ of sewage is discharged into the Mudan River trunk stream from those outlets; moreover, pollutants from some outlets are highly concentrated, even above the national sewage discharge standard and contaminate the river.

### 4.3. Model Parameters and Calibration Method

Model parameters that are required to be calibrated consist of horizontal diffusion coefficient, comprehensive decay rates of COD_Cr_ and NH_3_N as well as bed roughness under ice-covered and open-water period conditions. Some researchers suggest that erosion rate is a critical index affecting the precision of water quality simulation [37], but erosion rate is mainly influenced by particle size distribution, sediment bulk density, organic content, critical shear stress, and many external factors (*i.e.*, flow velocity, wind speed, vegetation). According to results reported by Qu *et al.* [38], there is little sediment runoff from the middle and lower reaches of the Mudan River trunk stream, and the river suspended load and the bed load are even less. For this reason, the proposed model did not take the influences of erosion rate on water quality into account.

Empirical values and trial methods can be brought together to define such parameters. In detail, empirical parameters calibrated with existing findings are firstly adopted and then adjusted, keeping the simulated results close to observed values; in the end, model parameters meeting simulation requirements are determined. Moreover, in order to guarantee model calculation convergence, a constant time step of 6-s was chosen for the simulation.

## 5. Validation of Hydrodynamics

As important parameters to characterize the size of resistances borne by river water bodies and one of the essential parameters for stream simulation and calculation, roughness coefficients reflects the impact of bed roughness on flow action. Under the condition of non-uniform flow in natural river courses, it is a coefficient under the combined actions of factors including stream equilibrium shape, watercourse hydraulic factor, section geometry and morphology, and bed surface characteristics and composition. The calculation of watercourse roughness mostly takes advantage of empirical or semi-empirical formulae. For watercourses in the open-water period, the roughness is computed by mainly taking the riverbed impacts into account. However, with respect to ice-covered rivers under the impact of ice sheets, boundary conditions for their free water surface turns into the ice sheet solid wall boundary condition; as vertical distributions of velocity are changed by ice sheets under the action of roughness, the velocity correspondingly decreases with the increase in watercourse roughness. For this research, roughness was in conformity with the results of Wang *et al.* [39], that is, 0.043 for the ice-covered period and 0.035 for the open-water period. In addition to the roughness coefficient, other parameters that need to be calibrated for the hydrodynamic process consist of the horizontal diffusion coefficients. After the findings of Chen *et al.* [40], Long *et al.* [41], Huang *et al.* [42] and HydroQual Inc. (Mahwah, NJ, USA) [43] values of these three coefficients of 1.0 m^2^/s, 1.0 × 10^−7^ m^2^/s and 1.0 × 10^−8^ m^2^/s, respectively, were used as references in this model.

**Figure 4:**
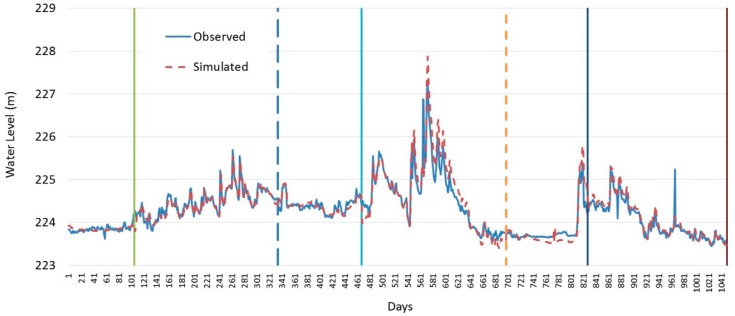
Result comparison between observed value and simulated value of water level at the Mudan River hydrological S2 station.

According to the simulation intervals defined in Table 1, hydrodynamic processes in ice-covered and open-water periods are simulated for the urban sections of the Mudan River trunk stream; furthermore, water level simulated results and observed water levels for the section at the Mudan River Hydrological S2 Station were compared with each other, as shown in Figure 4.

The figure suggests that during both the ice-covered and open-water periods, the model calculation results are in excellent agreement with the observed values, which indicates the water level variation process of simulated river are significant. During the ice-covered period, the measured mean water level is 224.04 m and the corresponding depth of water is 0.90 m; then, the depth of water corresponding to the simulated mean water level of 224.03 m is 0.89 m; the average error is only 0.01 m and the relative error of the average water depth is 1.11%. In comparison, during the open-water period, while the measured mean water level was 224.39 m with a corresponding depth of water of 1.25 m, the water depth corresponding to the average simulated water level of 224.42 m is 1.28 m, their average error is 0.03 m and their relative error of the average water depth is 2.40%. On the one hand, according to simulated velocity results for different water seasons, the velocity in the open-water period is significantly greater than that in the ice-covered period because the inflow in the open-water period is larger than that in the ice-covered period.

**Figure 5:**
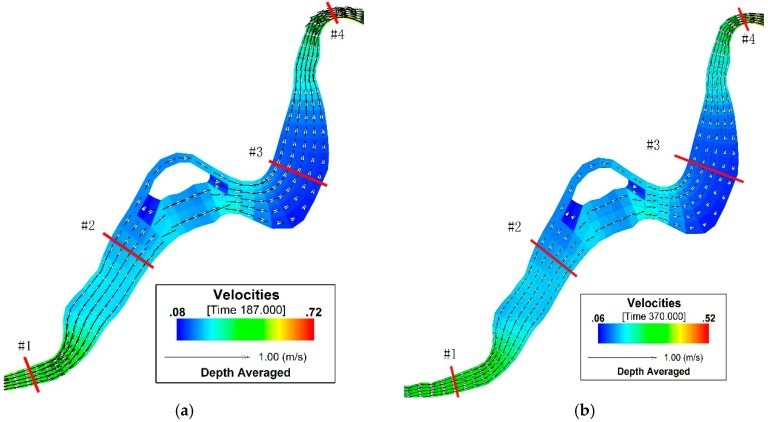
Schematic diagram for flow field in the urban section of Mudan River trunk stream. (**a**) open-water period; (**b**) ice-covered period.

Figure 5 reflects such a physical truth excellently. On the other hand, as observed from the aspect of flow field distribution characteristics, as the watercourse gently descends along the Xige section to the Chai River Bridge, the water flow direction is basically vertical to the watercourse section. Next to the center island of the watercourse, the flow direction changes due to its obstruction (Figure 5). Figure 5 shows the flow field simulated results on the 187th (high flow period) and 370th (dry season) days for the local channel in the research area. In this figure, the river widths of the #1 and #4 sections are 320 m and 181 m, respectively, and their flow velocities are correspondingly 0.43 m/s and 0.51 m/s on the 187th day while they are 0.29 m/s and 0.35 m/s on the 370th day. Concerning the #2 and #3 sections, their river widths are 813 m and 903 m; and the flow velocities on the 187th and the 370th days are 0.19 m/s and 0.12 m/s, and 0.14 m/s and 0.09 m/s. These results indicate that the flow velocity of the narrow reach is faster than that of the wide one, this is in conformity with physical truth of the research area.

## 6. Validation of Water Quality

### 6.1. Parameter Calibration Result Analysis

In the transport process during which pollutants enter into the river, concentration decay takes place by physical, chemical and biological actions, and the decay rates reflect the degradation velocities of those pollutants under the action of the water body. Currently, the parameter that needs to be calibrated by the overwhelming majority of water quality models is decay rate [44,45,46]. Through summarizing the findings of COD_Cr_ and NH_3_N decay rates in some rivers in China, it has been concluded by Guo *et al.* [47] that the COD_Cr_ decay rate for Chinese rivers is 0.009–0.470/day and it is 0.071–0.350/day for NH_3_N. Most of such rivers are however situated in warm areas with no or short ice-covered period.

According to simulation intervals defined in Table 1, transport process simulations were performed for COD_Cr_ and NH_3_N in simulated river reaches in ice-covered and open-water periods. Decay rates of COD_Cr_ and NH_3_N were 0.03/day and 0.05/day in the open-water period, and 0.01/day and 0.02/day for the ice-covered period. Compared with the decay rates of other rivers in China, mean values for COD_Cr_ and NH_3_N were 0.32/day and 0.23/day, respectively; COD_Cr_ and NH_3_N decay rates in the Mudan River are thus at a lower level during both the ice-covered and open-water periods; especially the decay rate of NH_3_N, which is even smaller than the minimum value (0.071/day) summarized by Guo *et al.* [47]. Considering possible reasons, they are related to the geographic position of the Mudan River which is further north than other rivers and also has a much lower multi-year average temperature [48,49].

According to the simulated results, decay rates of COD_Cr_ and NH_3_N in ice-covered period are both smaller than those in the open-water period. The main reasons are as follows: first, the water temperature in the ice-covered period is relatively low; and extremely low temperatures can directly affect the degradation of pollutants by microorganisms [50]. Second, due to the decreased inflow in the ice-covered period, the dilution effect of rivers on pollutants drained into them weakens; furthermore, together with the influence of ice sheets, the fluidity of the water body becomes poor so as to impact physical, chemical and biological reaction processes of pollutants [51,52]. Third, the water body is isolated from the atmosphere by ice sheets during the ice-covered period; consequently, reoxygenation due to natural aeration almost completely stops and the concentration of dissolved oxygen remains in a low state so that sources of dissolved oxygen required by organic matter degradation are restricted followed by a drop of the degradation rate [53].

### 6.2. Simulated Result Analysis

**Figure 6:**
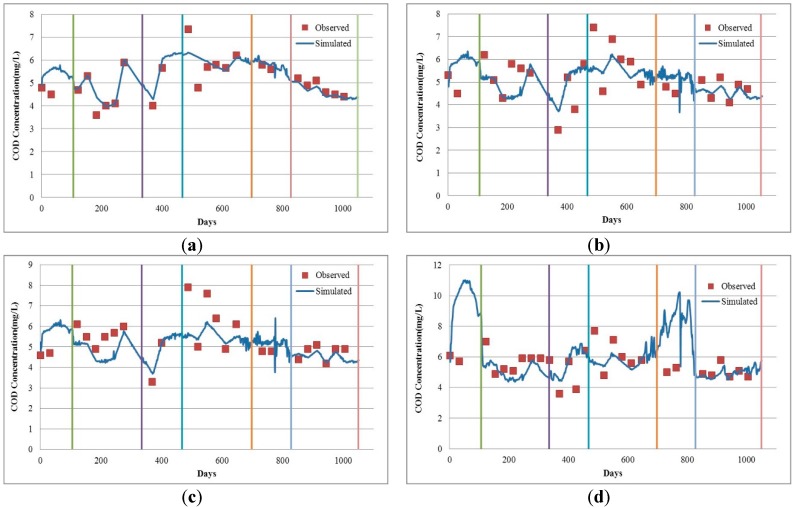
Result contrast charts for observed and simulated values of COD_Cr_ in the Mudan River trunk stream. (**a**) Wenchun Bridge; (**b**) Hailang; (**c**) Jiangbin Bridge; (**d**) Chai River Bridge.

Figure 6 compares the observed value and the simulated value of COD_Cr_ in the Mudan River trunk stream. It shows that variation trends of those two values are in good agreement. Statistical analysis results for the simulated and observed values of COD_Cr_ are shown in Table 2.

On the whole, from the perspective of the four verified sections, the Chai River Bridge one has the maximum average relative error of 18.43%, while the Wenchun Bridge section has the minimum (5.86%). Based on different simulation periods, the simulation effects of the four sections during the open-water period are better than those in the ice-covered period. To be specific, the simulation errors for the Wenchun and Jiangbin bridge section in both the ice-covered and open-water periods were 7.19% *vs.* 5.42% and 11.53% *vs.* 11.07%, respectively; in comparison, those for Hailang and Chai River Bridge in these periods were greatly different from each other, especially the average relative error of the latter in the ice-covered period is significantly larger than that in the open-water period (35.41% *vs.* 10.39%). The main reason causing such differences was insufficient observed values for the ice-covered period (Table 2). Moreover, the lack of concentration information in unmeasured months can exert an influence on the simulation effects of the ice-covered period.

**Figure 7:**
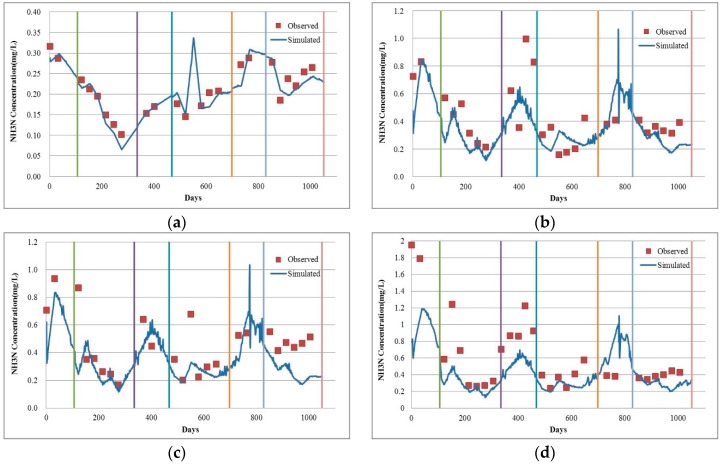
Result comparison for observed and simulated values of NH_3_N in the Mudan River trunk stream. (**a**) Wenchun Bridge; (**b**) Hailang; (**c**) Jiangbin Bridge; (**d**) Chai River Bridge.

As shown in Figure 7, a comparison of observed and simulated NH_3_N values in the Mudan River trunk stream, the NH_3_N simulation result can also be utilized to roughly reflect the actual variation situation. Table 3 presents the statistical analysis results for the simulated and observed values of NH_3_N in the Mudan River trunk stream model. As a whole, for the four verified sections, the Chai River Bridge has the maximum average relative error of 39.58% while that of the Wenchun Bridge is the minimum (14.88).

On the basis of the different simulation periods, unlike the COD_Cr_ simulation effect, there are differences in the simulation effects of these four sections during the open-water period. Among them, the simulation errors for the Wenchun and Jiangbin bridge sections in the ice-covered period are smaller than those in the open-water periods. However, such effects for Hailang and the Chai River Bridge in the open-water period are better than those in the ice-covered period. In general, it is feasible to apply the NH_3_N model to the Mudan River trunk stream despite the fact that its simulation accuracy is not as high as that of the COD_Cr_ simulation effects. The main cause for this is that the COD_Cr_ pollution source is primarily industrial discharge; continuous monitoring data are recorded for the concentration and discharge capacity of such pollutants and those data are able to support the model simulation. However, its NH_3_N pollution sources are mainly non-point source pollution and sanitary sewage within the drainage basin [54]. As only monitoring data of the sanitary sewage are noted down for the Mudan River trunk stream, which is lacking in non-point source pollution monitoring data, the accuracy of boundary conditions is lowered when it comes to the concentration of NH_3_N, so as to further exert an impact on the precision of our model simulation.

**Figure 8:**
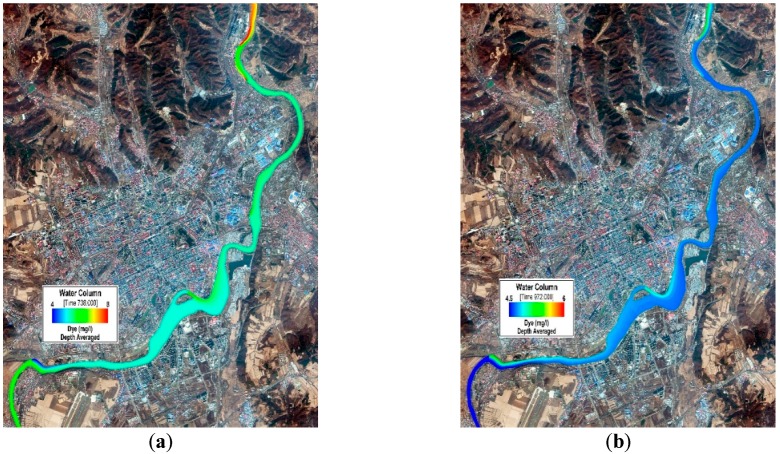
COD_Cr_ concentration distribution in the Mudan River trunk stream (urban sections). (**a**) 1-7-2014 (ice-covered period); (**b**) 8-29-2014 (open-water period).

**Figure 9:**
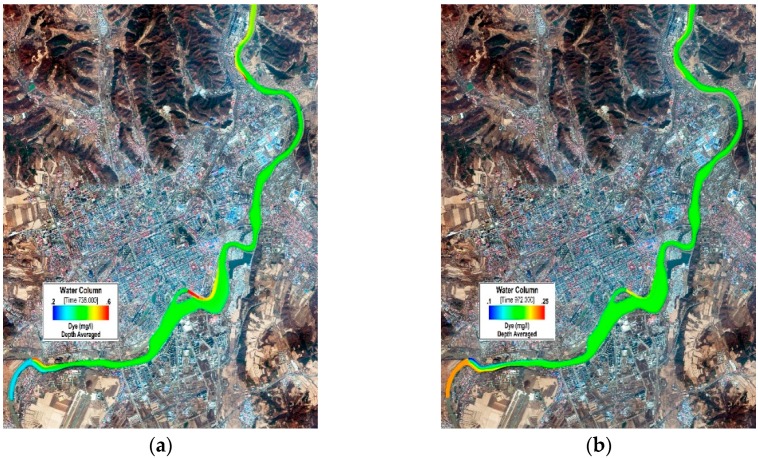
NH_3_N concentration distribution in the Mudan River trunk stream (urban sections). (**a**) 1-7-2014 (ice-covered period); (**b**) 8-29-2014 (open-water period).

Figure 8 and Figure 9 refer to the spatial distribution diagrams of the COD_Cr_ and NH_3_N simulated results in the ice-covered and open-water periods. On their basis, it can be clearly observed that as the concentration of pollutants discharged into the Mudan River from sewage outlets is intensively mixed after a certain distance under the action of attenuation and dispersion, sectional concentrations basically become consistent [55]. Basically a mixed concentration of COD_Cr_ or NH_3_N can be attained about 3 km to 5 km away from the sewage outlets where they are discharged. In case that a segment of watercourse in the downstream of sewage outlet is relatively straight, basically the mixed concentration can be reached after a long distance; while when such a segment undergoes twists and turns, the concentration can be mixed within a short distance under the impact of turbulence.

### 6.3. Problem Analysis

Although simulated values of COD_Cr_ and NH_3_N display excellent consistency with their observed values, certain errors still exist. It has been found that the main reasons leading to pollutant concentration calculation errors included the following aspects:

#### 6.3.1. Error of Bottom Elevation Generalization

With complex sectional form, a wide Mudan River reach often covers flood plains with different depths; therefore, its sections are usually in the shape of letters U or W. Given the stability of the model calculation, a watercourse was generalized as a rectangle in this model. In other words, the riverbed was deemed to be horizontal within cells. Due to such a generalization, some river sections, especially the W-shaped ones, can be distorted within a cell leading to simulation errors.

#### 6.3.2. Variability of Pollution Sources Discharged into the Watercourse from Urban Storm Drainage Pipe Networks and Sewage Pipes

Within the range of the simulated river reaches, there are large cities such as Mudanjiang City, Ning’an City and Hailin City, and some villages and towns including Wenchun Town, Hualin Town and Chaihe Town and so on are situated there too. Furthermore, along the river, there are many rainwater outflows and pollution discharge outlets whose discharge capacities and sewage concentrations mostly change dynamically. Especially the sanitary sewage outflow, and water discharges varied enormously during the different seasons. In this study, the adopted observed pollutant concentrations and wastewater discharges were measured quarterly. However, as data of some outflows were not monitored, the accuracy of the model flow and concentration boundary conditions declined.

#### 6.3.3. Dispersed Non-point Agricultural Source Pollution on Each Side of the Trunk Stream and Relevant Measurement Difficulties 

Water quality pollution in the Mudan River watershed is caused by the joint action of the point source pollution and the non-point source pollution [56]. Non-point source pollution sources within the Mudan River watershed mainly include urban non-point source pollution, irrigation backwater pollution, livestock breeding pollution and garbage pollution piled up disorderly in a rural area. In particular, the urban non-point sources refer to contamination of surface water, underground water and soil, caused by sanitary sewage and a small amount of industrial wastewater which are discharged into the river via a natural stream, an artificial stream or dispersed drainage; and such sanitary sewage and industrial wastewater may occur in multiple medium-and-small-sized towns and vast rural areas where no sewage treatment plants are established in the Mudan River region. Along the Mudan River, there are masses of farmlands. During irrigation, flood irrigation is adopted in most case so that a good deal of pesticides, chemical fertilizers and soil organic matter enter the river along with the irrigation backwater. In addition, there is large-scale livestock and poultry breeding along the Mudan River, but pollution prevention measures are inadequate among most of the breeding industries. Consequently, faeces produced by livestock breeding are piled up at random and it is extremely easy for them to be washed away in huge quantities. Especially during the rainy season, part of them seep into the underground and may contaminate the ground water; and the other part can flow into nearby rivers, lakes and reservoirs along with rainwater resulting in organic pollution of the surface water. Moreover, sufficient waste treatment plants have not been constructed in different towns along the Mudan River watershed. Garbage is randomly piled up on both sides of the watercourse; after they enter a water body due to rainwash, and it can be contaminated directly. Considering that those pollution sources are very scattered and hard to control, parameter calibration and verification of the model is very challenging.

## 7. Conclusions

In this work, a new two-dimensional hydrodynamic water quality model has been established to simulate COD_Cr_ and NH_3_N concentrations during the ice-covered and open-water periods for the Mudan River in China on the basis of EFDC. Moreover, we calibrated and verified the parameters involved, including the dispersion coefficient, the riverbed roughness and the comprehensive decay rate. Through analysis of our research, some conclusions can be drawn as follows:

(1) Research findings show that the concentration simulation errors of COD_Cr_ in the four sections used for model verification range from 5.86% to 18.43%; while for those of NH_3_N, are between 14.88% and 39.58%.
(2) The decay rate of COD_Cr_ and NH_3_N during the ice-covered period is lower than that in the open-water period. According to the research results, in the trunk stream of the Mudan River, the decay rates of COD_Cr_ and NH_3_N during the open-water period are 0.03/day and 0.05/day while they are 0.01/day and 0.02/day during the ice-covered period.
(3) For this research, the roughness adopted for the ice-covered period was 0.043 and 0.035 for the open-water period. The obtainment of favorable simulation effects indicates that these two parameters were selected appropriately.
(4) Comparing with the decay rates of other rivers in China, those of COD_Cr_ and NH_3_N in the Mudan River are relatively lower. This may be due to the low annual average temperature of this river which is located in the cold north region.
(5) Within the research area, due to the lack of measured data about the non-point source pollution sources, the NH_3_N simulation precision is lower than that of COD_Cr_, so monitoring of the non-point source pollution should be enhanced for the Mudan River watershed in order to fully grasp the NH_3_N pollution pattern and further improve the NH_3_N simulation precision.

## Figures and Tables

**Table 1:** Simulation intervals for hydrodynamic water quality model of the Mudan River trunk stream.

Simulation Interval (Days)	Number of Days	Water Season	Simulation Interval (Days)	Number of Days	Water Season
1–106	106	ice-covered period	107–335	229	open-water period
336–468	133	ice-covered period	469–698	230	open-water period
699–828	130	ice-covered period	829–1050	222	open-water period

**Table 2:** Statistical results for COD_Cr_ simulated and observed values in the Mudan River trunk stream model.

Simulation Period	Wenchun Bridge		Hailang		Jiangbin Bridge		Chai River Bridge	
	Sample Size	Average Relative Error (%)	Sample Size	Average Relative Error (%)	Sample Size	Average Relative Error (%)	Sample Size	Average Relative Error (%)
ice-covered period	6	7.19	8	17.44	6	11.53	9	35.41
open-water period	18	5.42	18	10.66	18	11.07	19	10.39
in total	24	5.86	26	12.75	24	11.18	28	18.43

**Table 3:** Statistical analysis for simulation and observed values of NH_3_N in the Mudan River trunk stream model.

Simulation Period	Wenchun Bridge		Hailang		Jiangbin Bridge		Chai River Bridge	
	Sample Size	Average Relative Error (%)	Sample Size	Average Relative Error (%)	Sample Size	Average Relative Error (%)	Sample Size	Average Relative Error (%)
ice-covered period	6	10.92	8	35.94	6	21.66	9	55.15
open-water period	17	16.28	17	33.42	18	35.35	19	32.32
in total	23	14.88	25	34.23	24	31.93	28	39.58
